# Comparison of WAIC and posterior predictive approaches for N-mixture models

**DOI:** 10.1038/s41598-024-66643-4

**Published:** 2024-07-08

**Authors:** Heather E. Gaya, Alison C. Ketz

**Affiliations:** 1 Warnell School of Forestry and Natural Resources, University of Georgia, Athens, GA 30602 USA; 2 Wisconsin Cooperative Research Unit, Department of Forest and Wildlife Ecology, University of Wisconsin, Madison, WI 53706 USA

**Keywords:** Bayesian, eBird, Model selection, N-mixture, Posterior-predictive loss, WAIC, Ecology, Ecological modelling, Statistical methods

## Abstract

Hierarchical models are common for ecological analysis, but determining appropriate model selection methods remains an ongoing challenge. To confront this challenge, a suitable method is needed to evaluate and compare available candidate models. We compared performance of conditional WAIC, a joint-likelihood approach to WAIC (WAICj), and posterior-predictive loss for selecting between candidate N-mixture models. We tested these model selection criteria on simulated single-season N-mixture models, simulated multi-season N-mixture models with temporal auto-correlation, and three case studies of single-season N-mixture models based on eBird data. WAICj proved more accurate than the standard conditional formulation or posterior-predictive loss, even when models were temporally correlated, suggesting WAICj is a robust alternative to model selection for N-mixture models.

## Introduction

Model selection is a critical part of ecological inference^1–3^, allowing scientists to weigh and ultimately choose between competing ecological hypotheses. In the absence of clear empirical evidence or infinite time to perform manipulative experiments, computational model fitting allows researchers to weigh the merits of alternative models^1,4,5^. However, despite an increase in the use of Bayesian models in recent decades^6,7^, rigorously tested methods of Bayesian model selection have remained relatively sparse^5^.

Uncertainty in ecological models comes from two main sources, ecological variability and sampling bias. First, the ecological process model reflects variation in the state of the system itself^8^. Further, uncertainty in the ecological process also reflects mechanistic uncertainty in the environmental variables for predicting system outcomes^3^. Second, observations of ecological data are never perfect and direct information on the system is rarely available^9^. Observations are often biased by imperfect detection, imprecision in measurement tools, and human error. To confront these challenges, a suitable method is needed to evaluate and compare available candidate models, while accounting for uncertainty.

One common method used for Bayesian model selection is the Watanabe-Akaike Information Criteria (WAIC)^10^, a generalized version of AIC. WAIC combines the estimated log predictive density of the data, with a penalty for the estimated number of parameters in the model to produce a value that can be compared between alternative models. This criteria is valid in both hierarchical and mixture models^10^ and has been shown to be an efficient alternative to cross-validation when data are uncorrelated^11,12^. Mathematically, WAIC only utilizes the fit of the data to the observation process, suggesting this method may not be appropriate for discriminating between alternative models with different underlying state processes. Moreover, WAIC is only appropriate when processes do not have spatial or temporal auto-correlation^5^, which is an assumption rarely met for ecological systems.

We evaluate 3 prediction-based Bayesian model selection approaches that can be used to evaluate models when there is auto-correlation in the ecological process and imperfect detection in the observation process. We compare performance of conditional WAIC, posterior-predictive loss and a joint-likelihood approach to WAIC that accounts for the joint-likelihood of both the observation and state processes. We test these model selection criteria on simulated single-season N-mixture models, simulated multi-season N-mixture models with temporal auto-correlation, and three case studies of single-season N-mixture models for Bewick’s wren (*Thryomanes bewickii*), mourning dove (*Zenaida macroura*) and the house finch (*Haemorhous mexicanus*) based on eBird data^13^ collected in California.

## Methods

### WAIC

WAIC^10^ is calculated using the expected log predictive densities for all data points. Following^11^, WAIC is defined as:

1 $$\begin{aligned} WAIC = -2\widehat{lpd} + 2\widehat{p}_{waic} \end{aligned}$$

where $\widehat{lpd}$ is the estimated log predictive density of the data $(\widehat{lpd} = \sum _{i = 1}^{n} \log (P(y_i | \hat{\theta _i})))$, and $\widehat{p}_{waic}$ is the estimated effective number of parameters $(\widehat{p_{waic}} = \sum _{i = 1}^{n} var_{post}(\log (P(y_i | \hat{\theta _i})))$. If the posterior distribution of $\theta$ is obtained directly from the likelihood of $y_i$ without any latent processes, and there is no sampling bias in the observation process, then WAIC is sufficient to identify the top model amongst all candidate models. However, in many ecological studies, sampling error is introduced because ecological processes are inherently difficult, if not impossible, to observe completely. Ecologists can address imperfect detection during model development and selection.

We demonstrate the impact of imperfect detection on model selection using a Binomial *N*-mixture model, where both abundance (*N*) and detection probability (*p*) vary with site *i*. Detection probability may also vary between surveys (*j*) due to weather or other temporal environmental conditions. The data, $y_{ij}$ are the observed abundance at site *i* during survey *j* of ($1 \cdots J$) surveys.

2 $$\begin{aligned} \mathrm {\log }(\mu _i)&= \beta _0 + \beta _1 x_{i1} + \beta _2 x_{i2} + \cdots \end{aligned}$$

3 $$\begin{aligned} N_i&\sim \textrm{Poisson}(\mu _i) \end{aligned}$$

4 $$\begin{aligned} \textrm{logit}(p_{ij})&= \alpha _0 + \alpha _1 x_{i1} + \cdots \end{aligned}$$

5 $$\begin{aligned} y_{ij}&\sim \textrm{Binomial}(N_i, p_{ij}). \end{aligned}$$

Traditionally, WAIC does not directly use the posterior of $N_i$, the state process. As $p_{ij}$ approaches 1, the log predictive density of the data approaches 0 and the state process has no effect on WAIC. Thus when $p_{ij}$ approaches 1, WAIC cannot distinguish differences in the fit of candidate models with different covariates on *N*.

6 $$\begin{aligned} P(y_{ij}| p_{ij}, N_{i})&= \left( {\begin{array}{c}N_i\\ y_{ij}\end{array}}\right) p_{ij}^{y_{ij}}(1-p_{ij})^{(N_i-y_{ij})} \end{aligned}$$

7 $$\begin{aligned} \lim _{p_{ij} \rightarrow 1} \log (P(y_{ij}| p_{ij}, N_{i}))&= \left( {\begin{array}{c}N_i\\ y_{ij}\end{array}}\right) 1^{y_{ij}}(0)^{(N_i-y_{ij})} = 0 \end{aligned}$$

Similarly, as $p_{ij}$ approaches 0, the log predictive density of the data also approaches 0:

8 $$\begin{aligned} \lim _{p_{ij} \rightarrow 0} \log (P(y_{ij}| p_{ij}, N_{i})) = \left( {\begin{array}{c}N_i\\ y_{ij}\end{array}}\right) 0^{y_{ij}}(1)^{(N_i-y_{ij})} = 0 \end{aligned}$$

In other words, if the parameter accounting for imperfect detection is near the boundaries of its distribution, the appropriate use of WAIC is limited.

### Posterior predictive loss

We also consider a largely untested model selection method, posterior predictive loss^14,15^, that is theoretically appropriate for model selection when there is auto-correlation in the ecological process. Posterior predictive loss ($D_{sel}$) rewards models that minimize the difference between expected $\mu _{ij}$ and observed data $y_{ij}$ (i.e., model fit) by minimizing the predictive variance ($\widehat{\sigma ^2}$). It is analogous to familiar information criteria, but does not penalize models based on the effective number of parameters^5^. Posterior predictive loss is defined as

9 $$\begin{aligned} D_{sel} = \sum _{i = 1}^{n} (\mu _{ij} - y_{ij}) + \sum _{i = 1}^{n} {\sigma ^2_{i}}, \end{aligned}$$

where $\mu _{ij} = E(y_{ij})$ and $\sigma ^2_{i}$ is the posterior variance of $y_{ij}$. Similar to WAIC, posterior predictive loss only considers the observation process in the likelihood of $y_{ij}$ and does not incorporate direct inference on latent processes. In other words, changes to the modeling structure for *N* (e.g. adding or removing covariates) are not directly captured by posterior predictive loss.

### WAICj

We developed a joint-likelihood adjustment to WAIC based on the joint distribution of the observation and latent processes. We define our joint-likelihood approach to WAIC as:

10 $$\begin{aligned} WAICj =&-2\widehat{\lambda _{\text {j}}} + 2\widehat{p_{waicj}}, \end{aligned}$$

11 $$\begin{aligned} \lambda _{\text {j}} =&\sum _{i = 1}^{n} \log (P(y_i | N_i)) + \log (P(N_i | \mu _i)) \end{aligned}$$

12 $$\begin{aligned} p_{waicj} =&\sum _{i = 1}^{n} var_{post}(\log (P(y_i | N_i)) + \log (P(N_i | \mu _i))). \end{aligned}$$

where $\mu _i$ is the expected abundance at site *i* given $N_i \sim \textrm{Poisson}(\mu _i)$. Unlike the traditional formulation of WAIC, the log predictive density of the data does not approach 0 when $p_{ij}$ reaches the boundaries of its distribution.

13 $$\begin{aligned} \lim _{p_{ij} \rightarrow 1} \log (P(y_i | N_i)) + \log (P(N_i | \mu _i))&= \frac{{\mu _{i}}^N_{i} e^{-\mu _i}}{N_i!} \end{aligned}$$

Under this formulation, model selection accounts for the log likelihood in both the detection model and the underlying state process model. Thus, if abundance is thought to vary between sites in response to environmental covariates, model selection between candidate models with different covariates should be possible, even as detection probability approaches the boundaries of its distribution.

### Simulation—single season N-mixture models

We simulated abundance in an N-mixture model framework for 300 data sets to compare these 3 model selection approaches including the standard conditional WAIC, posterior predictive loss, and our proposed joint-likelihood WAIC (WAICj). We considered our simulations to be analogous to a survey of bird populations. We generated a covariate representing the proportion of forested area at each site, and then simulated ‘bird’ abundance at 50 sites as a random draw from an inhomogenous Poisson based on the simulated covariate. The coefficient for the covariate ($\theta _i$) was randomly drawn for each simulation and then we generated the state process model ($N_i$). We simulated detection as four independent visits of each site assuming a single covariate effect on detection probability that would be analogous to wind speed.

We evaluated model selection performance under three different sampling intensities (15, 25 or 50 sites) and two detection scenarios (low detection and high detection). We specified the low detection scenario to have an average detection probability of 0.13 (0.06–0.24). The high detection probability scenario had an average detection probability of 0.84 (0.73–0.93). We analyzed generated data from each simulation using 4 candidate binomial N-mixture models (Table 1) and used all three formulations of model selection criteria to assess model performance. For each criteria, we calculated the weight of each candidate model following the standard formula for Akiake weights^1,16^. We evaluated the performance of model selection criteria by calculating the proportion of simulations where the model that was selected was the true generating model.

**Table 1:** Candidate models. The asterisk denotes the model under which data was simulated. Periods represent intercept only models.

Model number	Formulation
Model 1*	$\mu (forest_i)p(wind_{it})$
Model 2	$\mu (.)p(.)$
Model 3	$\mu (forest_i)p(.)$
Model 4	$\mu (.)p(wind_{it})$

We also assessed performance when additional site visits were added for each sampling intensity. Using the same sampling intensities and detection scenarios as described above, we simulated data from 4, 8, and 16 independent visits to each site. We evaluated the performance of model selection criteria by calculating the proportion of simulations where the model that was selected was the true generating model.

### Simulation—multi-season N-mixture models

We also simulated abundance under a dynamic N-mixture to compare the 3 model selection approaches when temporal auto-correlation was present in abundance. The model for first year abundance ($N_{i1}$) matched the single-season scenarios, with abundance dependent on a simulated site-level covariate ($x_{i1}$) representing forest cover.

14 $$\begin{aligned} \mathrm {\log }(\mu _{i1})&= \beta _0 + \beta _1 x_{i1}, \end{aligned}$$

15 $$\begin{aligned} N_{i1}&\sim \textrm{Poisson}(\mu _{i1}) \end{aligned}$$

Following the first year, abundance at each site was simulated as a function of the previous year’s expected abundance and a site-specific growth rate, dependant on the site-level covariate and an effect of time period (*t*).

16 $$\begin{aligned} \mathrm {\log }(\psi _{it})&= \delta _0 + \delta _1 x_{it} + \delta _2 t \end{aligned}$$

17 $$\begin{aligned} \mu _{it}&= \psi _{it} \mu _{it-1} \end{aligned}$$

18 $$\begin{aligned} N_{it}&\sim \textrm{Poisson}(\mu _{it}) \end{aligned}$$

We simulated the multi-season model with two time trends, weak and strong. As with the single season N-mixture models, we evaluated model selection performance under two detection scenarios (low detection and high detection) and when 15, 25 or 50 sites were sampled with 4 independent visits per site. For each scenario we simulated 100 data sets, each with 5 years of data, for a total of 600 data sets. We used the same observation process and candidate models as in the single-season scenarios.

### Case study—California eBird data

We applied N-mixture models to point count data for three species of birds in California, U. S., to demonstrate the effect of model selection outcomes. We used data from the global eBird database that consisted of semi-structured avian point count data collected by community scientists^17^. Data are reported as ‘checklists’ of species detections, with the location, time of survey, number of observers, length of survey, and number of detections of each species reported for each checklist. We selected 180 eBird ‘complete checklists’ recorded across 48 locations in southern California between April and July, 2019. We chose these dates to represent the breeding season, when male birds may be more likely to vocalize. Data were downloaded from eBird and formatted using the auk package in R^13,18^ and code presented in Goldstein^19^.

We focused our analysis on three bird species with differing numbers of total raw counts—Bewick’s wren, mourning dove and the house finch. For each site, we considered 3 possible environmental covariates that could potentially affect abundance (elevation, proportion of forest cover and proportion of water) and 4 detection covariates (duration of survey, number of observers, proportion of forest cover and proportion of water). Elevation and landcover data for the state of California were downloaded from WorldClim^20^ and LandFire GIS database’s Existing Vegetation Type layer^21^ respectively. For each species, we fit 16 candidate binomial N-mixture models and used the three model selection criteria to assess model performance.

All simulations and case study models were approximated using Markov-chain Monte Carlo using the nimble package^22^ in R version 4.1.3^23^. We used 2 MCMC chains with 10,000 iterations and a thinning rate of 1. Chain convergence was assessed by the Gelman-Rubin statistic^11^ and visual inspection of traceplots.

## Results

**Figure 1:**
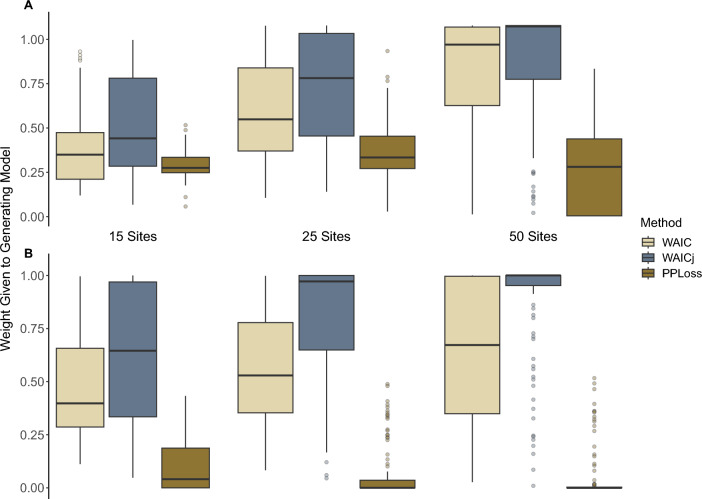
Weight given to the generating model when simulated detection probability was close to 0 (**A**) or approaching 1 (**B**). Each simulation scenario was analyzed 100 times with data from 15, 25 or 50 sites.

When applied to single-season N-mixture models, the WAICj approach ranked the correct model as the top model more often than the other model selection methods (Table 2), and identified the correct top model in 74.2% of the simulations. Standard conditional WAIC correctly identified the top model in 59.3% of the simulations. Model selection using posterior predictive loss performed poorly under all scenarios and only identified the correct top model in 10.3% of simulations. There was no difference in performance between the standard WAIC and WAICj approaches for the low detection probability scenario. For the simulated single-season N-mixture models, increased sample size led to more accurate model selection for both the standard WAIC and WAICj approaches (Fig. 1).

**Table 2:** Proportion of simulations (n = 100) where the generating model was chosen as the top ranked model.

	No. sites		
	15	25	50
Single-season			
Low detection			
WAIC	0.44	0.67	0.83
WAICj	0.53	0.69	0.82
PP Loss	0.19	0.24	0.11
High detection			
WAIC	0.43	0.57	0.62
WAICj	0.68	0.82	0.91
PP Loss	0.02	0.03	0.03
Weak time-trend			
Low detection			
WAIC	0.91	0.88	0.85
WAICj	0.90	0.94	0.92
PP Loss	0.13	0.08	0.15
High detection			
WAIC	0.54	0.44	0.38
WAICj	0.91	0.92	0.93
PP Loss	0.03	0.01	0.00
Strong time-trend			
Low detection			
WAIC	0.99	0.98	0.96
WAICj	0.97	0.95	0.86
PP loss	0.45	0.53	0.54
High detection			
WAIC	0.92	0.92	0.98
WAICj	0.99	0.98	1.00
PP loss	0.38	0.43	0.46

When we increased the number of simulated visits per site, both the standard WAIC and WAICj tended to produce more accurate results as visits increased, regardless of the detection scenario (Fig. S1). Posterior predictive loss showed a similar pattern to WAIC when detection was close to 1, but showed a drop in performance with increased visits when detection was close to 0. Regardless of the number of site visits, WAICj was the most accurate of the three tested methods when identifying the generating model.

**Figure 2:**
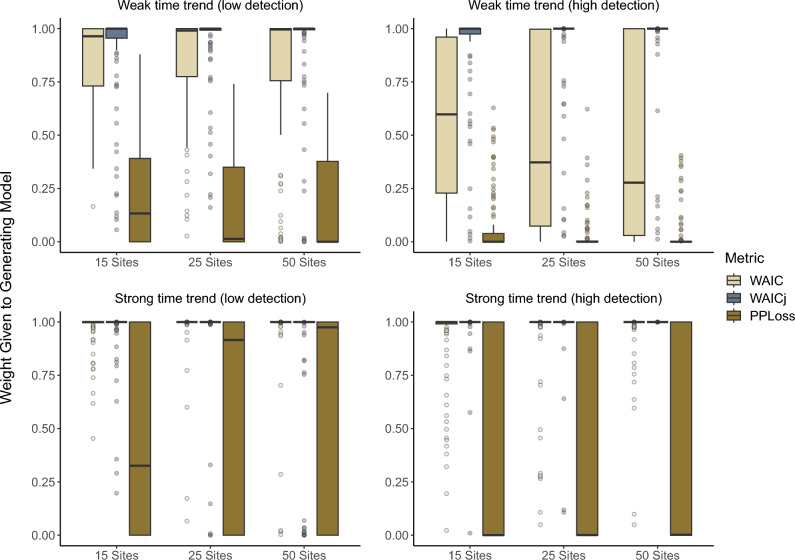
Weight given to the generating model when a weak or strong time trend was present in abundance and detection probability was close to 0 (low detection) or approaching 1 (high detection). Each scenario was analyzed 100 times with data from 15, 25 and 50 sites.

**Figure 3:**
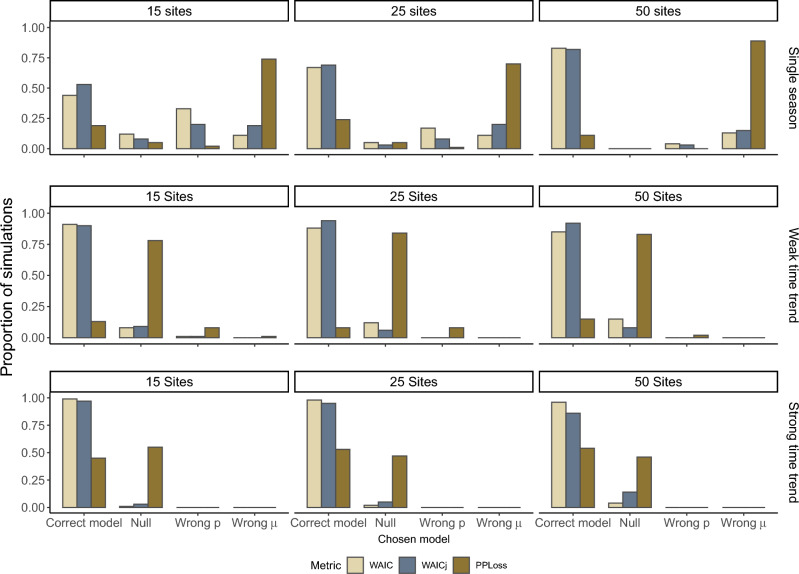
Proportion of simulations in which each alternative model was selected as the top model when no time trend, a weak time trend or a strong time trend was present in abundance and detection probability was approaching 0 (low detection scenario). Each scenario was analyzed 100 times with data from 15, 25 and 50 sites.

For the multi-season models with time trends, WAICj correctly identified the generating model 86–100% of time (Table 2). WAIC and WAICj weighted the generating model close to 1 when the generating model was identified as the top model (Fig. 2). The joint likelihood approach consistently performed better under high detection for both the weak time trend and strong time trend scenarios. Conditional WAIC identified the correct model in 45% of simulations with a weak time trend when detection was high, but identified the correct model in 98–99% of simulations when there was a strong time trend present. Posterior predictive loss correctly chose the generating model in 0–47% of the time-trend scenarios (Fig.  3).

Between April and July, 2019, 24 Bewick’s wren, 101 mourning dove and 350 house finch were detected across 180 eBird checklists at 48 locations. Across the three species, model selection results were inconsistent (Table 3). Bewick’s wren and mourning dove had conflicting results for the top model based on the three methods. For the house finch, the conditional and joint-likelihood approaches to model selection consistently supported the same top model. For all three species, posterior predictive loss indicated varying top models compared with the other model selection approaches.

**Table 3:** Model weight given to the top 3 candidate models for three bird species detected by eBird surveys in southern California from April to July, 2019.

	Abundance model	Detection model	Weight
Bewick’s Wren			
WAIC	$\mu (elevation + forest + water)$	$p(duration + observers + forest + water)$	0.27
	$\mu (elevation + forest)$	$p(duration + observers + forest + water)$	0.24
	$\mu (elevation + forest + water)$	$p(duration + observers + forest)$	0.17
WAICj	$\mu (elevation + forest)$	$p(duration + observers + forest + water)$	0.96
	$\mu (elevation + forest + water)$	$p(duration + observers + forest + water)$	0.04
	$\mu (elevation + forest + water)$	$p(duration + observers + forest)$	0.00
PP Loss	$\mu (elevation)$	$p(duration + observers + forest)$	0.19
	$\mu (elevation + forest)$	$p(duration + observers + forest)$	0.18
	$\mu (elevation + forest)$	$p(duration + observers + forest + water)$	0.16
Mourning Dove			
WAIC	$\mu (elevation + forest)$	$p(duration + observers + forest)$	0.24
	$\mu (elevation + forest + water)$	$p(duration + observers + forest + water)$	0.15
	$\mu (elevation + forest + water)$	$p(duration + observers + forest)$	0.14
WAICj	$\mu (elevation + forest + water)$	$p(duration + observers + forest)$	1.00
	$\mu (elevation + forest)$	$p(duration + observers + forest)$	0.00
	$\mu (elevation + forest + water)$	$p(duration + observers + water)$	0.00
PP Loss	$\mu (elevation + forest)$	$p(duration + observers + forest + water)$	0.24
	$\mu (elevation + forest)$	$p(duration + observers + forest)$	0.23
	$\mu (elevation + forest + water)$	$p(duration + observers + forest + water)$	0.12
House Finch			
WAIC	$\mu (elevation + forest + water)$	$p(duration + observers + forest + water)$	1.00
	$\mu (elevation + forest + water)$	$p(duration + observers + water)$	0.00
	$\mu (elevation)$	$p(duration + observers)$	0.00
WAICj	$\mu (elevation + forest + water)$	$p(duration + observers + forest + water)$	1.00
	$\mu (elevation + forest + water)$	$p(duration + observers + water)$	0.00
	$\mu (elevation + water)$	$p(duration + observers + forest + water$	0.00
PP Loss	$\mu (elevation)$	$p(duration + observers + forest)$	0.72
	$\mu (elevation)$	$p(duration + observers)$	0.28
	$\mu (elevation + forest + water)$	$p(duration + observers + forest + water)$	0.00

**Figure 4:**
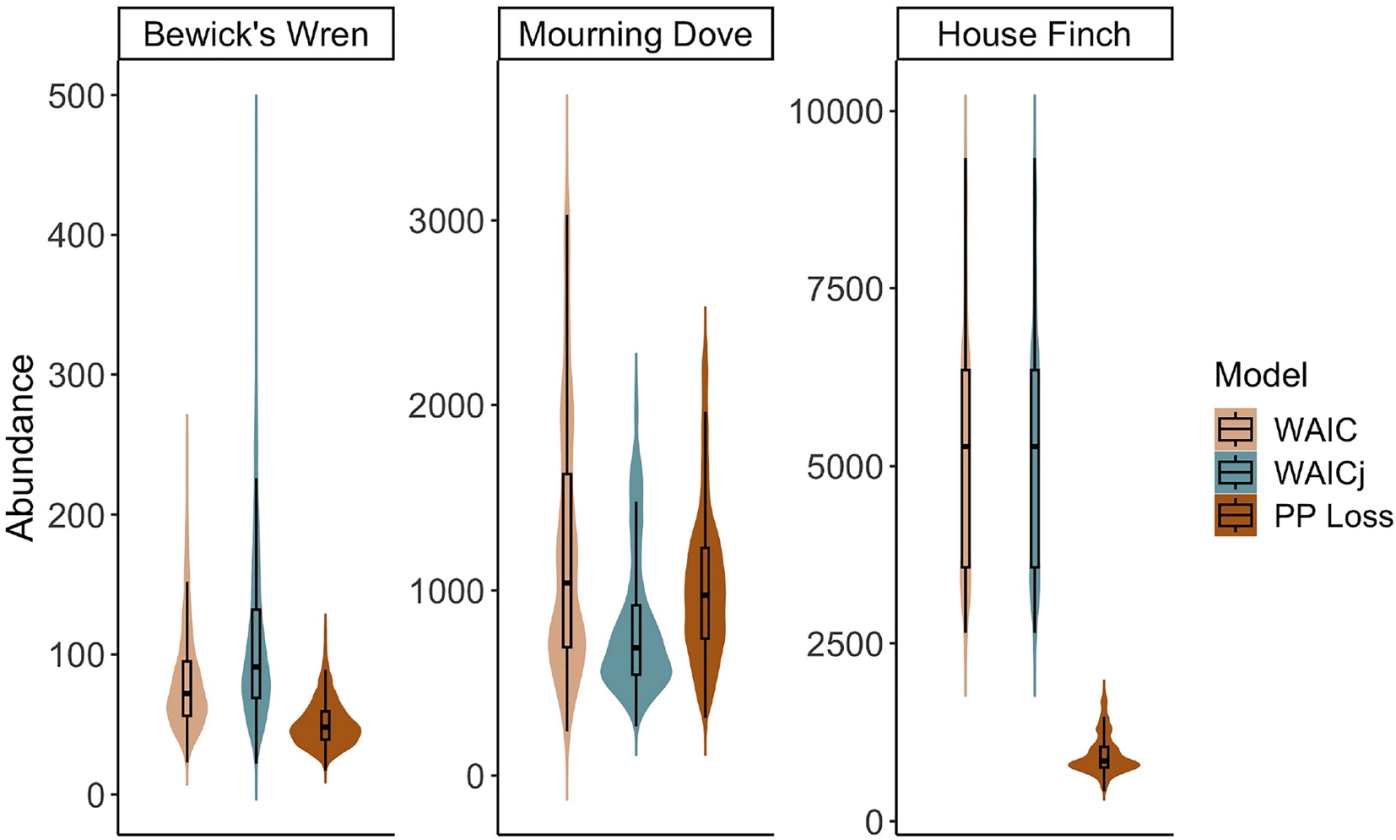
Posterior distributions of abundance for three bird species detected by eBird surveys in southern California from April to July, 2019. Violin plots show the posterior distribution from the top model selected by the traditional, joint-likelihood or posterior predictive loss approaches to WAIC.

Despite differences in model ranking, there was little difference in the overall estimated abundance for Bewick’s wren or mourning dove based on the top model selected by each method (Fig. 4). However, the top model for house finch selected by both the conditional and joint-likelihood approaches to WAIC suggested a substantially higher abundance than the model selected by the posterior predictive loss approach. We did not evaluate goodness of fit for any of the models, as this was outside the scope of the study.

## Discussion

Model selection is critical for discriminating between alternative ecological hypotheses^4,6^. Hierarchical models are now ubiquitous in ecology, but determining appropriate model selection methods remains an ongoing challenge^2,5,24^. WAIC methods are used analogously for hierarchical Bayesian models, much like AIC is applied in maximum likelihood analyses, especially when data are plentiful and detection probability is moderate^5,11^. However, we found that the conditional calculation of WAIC was inconsistent under low data scenarios and when detection parameters were approaching the boundaries of their distributions. The joint likelihood formulation of WAIC (WAICj) proved more accurate than the standard conditional formulation, even when models were temporally correlated, suggesting WAICj is a robust alternative to model selection.

We expected increased sampling intensity (larger number of sites) to improve model selection accuracy, but the results were inconsistent across simulation scenarios and between selection methods. In both the single season and weak time trend scenario, posterior predictive loss had an inverse relationship between accuracy (correctly identifying the top model) and the number of sites in the dataset. This did not correspond to a lack of data, as we saw the same behavior when additional sites were added to our single season high detection scenario. Posterior predictive loss prioritizes the fit of the observed data to the observation model, as demonstrated by the increased selection of the incorrect process model (model 4) as sampling intensity increased. Thus, when two candidate models have similar posterior distributions according to the observation process, the penalty terms are also similar, leading to inaccurate conclusions^25^. This behavior suggests that large sample size alone does not guarantee accuracy in model selection, particularly when fit of the process model is not directly incorporated in model selection calculations.

Despite improvements in model selection accuracy when compared to posterior predictive loss and the traditional formulation of WAIC, WAICj showed poor performance when evaluating single-season N-mixture models with a low number of sites. We suspect this poor performance stems in part from the N-mixture modeling structure. One drawback to N-mixture models is the lack of information contained in repeated, unmarked counts. When the number of sites visited or the number of independent visits to each site are low, model parameters may not be clearly identifiable^26,27^. As Barker et al.^28^ demonstrated using AIC, sparse data generated under an N-mixture model is practically indistinguishable from data generated under alternative models when detection probability is constant (or close to constant). Therefore, the appropriate use of WAICj is naturally limited to sample sizes that are sufficiently large to produce fully identifiable models.

When applied to eBird data, the three model selection approaches tended to select different top models. While conditional and joint likelihood approaches to WAIC always agreed on the observation process and had similar estimates for total abundance, posterior predictive loss consistently chose alternative candidate models. Notably, the top model for house finch selected by posterior predictive loss suggests approximately 4000 fewer individuals than the top models selected by WAIC and WAICj. In conjunction with the simulation results, this deviation likely reflects the unsuitability of posterior predictive loss for discriminating between alternative N-mixture models. Moreover, the inconsistencies between the top models selected by each selection criteria highlight the potential for poor model selection methods to lead to inaccurate inference on the ecological relationships between a species of interest and its environment.

Posterior predictive loss performed unreliably across all simulation scenarios, and we caution ecologists against using this metric for model selection. Previous research has suggested that posterior predictive loss can be an effective model selection method when data are observed without error^15^ and should be suitable for correlated data^5^. However, consistency of posterior predictive based criteria appears to be dependent on the model structure^25^ and may perform poorly when data are only partially observed^29^. While we cannot unilaterally conclude that posterior predictive loss is inappropriate for all model selection endeavors, we do not recommend this approach for discriminating between alternative N-mixture models.

Previous research has shown that WAIC is inappropriate for model selection for correlated data, especially for spatial models such as spatial capture-mark recapture^2,5^. An underlying assumption in the calculation of WAIC is independence of the data given the parameters, an assumption that was violated in the multi-season simulations. The conditional calculation of WAIC performed inconsistently when a time-trend was present in the model, reflecting that the violations of the assumption of independence is problematic for this information criteria. However, we found that WAICj was a valid choice for model selection even when data were temporally correlated. Based on these results, we suggest that WAICj may be appropriate for both single season and multi-season N-mixture models, but suggest caution when applying this model selection criteria outside of an N-mixture modeling framework. Further research could address the utility of our joint WAIC approach developed here, for other hierarchical Bayesian models.

Model selection methods, even for relatively simple hierarchical models, requires more development to produce consistent and reliable results. WAICj produced more consistent results than conditional WAIC or posterior predictive loss, but all of the tested model selection criteria performed poorly in at least one simulation scenario. For many ecological studies, detection probability is often low^9^ and extensive sampling can be cost-prohibitive. Thus, while N-mixture models may offer a flexible solution for modeling count data, we suggest caution when performing model selection and urge ecologists to carefully consider the weaknesses of their chosen model selection method when evaluating ecological hypotheses.

### Supplementary Information

Supplementary Information.

## Data Availability

All scripts and data necessary to run the analysis are publicly available: https://zenodo.org/records/10680420
