# Supplementary material for: Comparison of WAIC and posterior predictive approaches for N-mixture models
Source: Sci Rep. 2024 Jul 8;14:15743. doi: 10.1038/s41598-024-66643-4 (PMC11231229; doi:10.1038/s41598-024-66643-4)
Supplement: Supplementary file 1 — Supplementary Information. [file 41598_2024_66643_MOESM1_ESM.pdf]

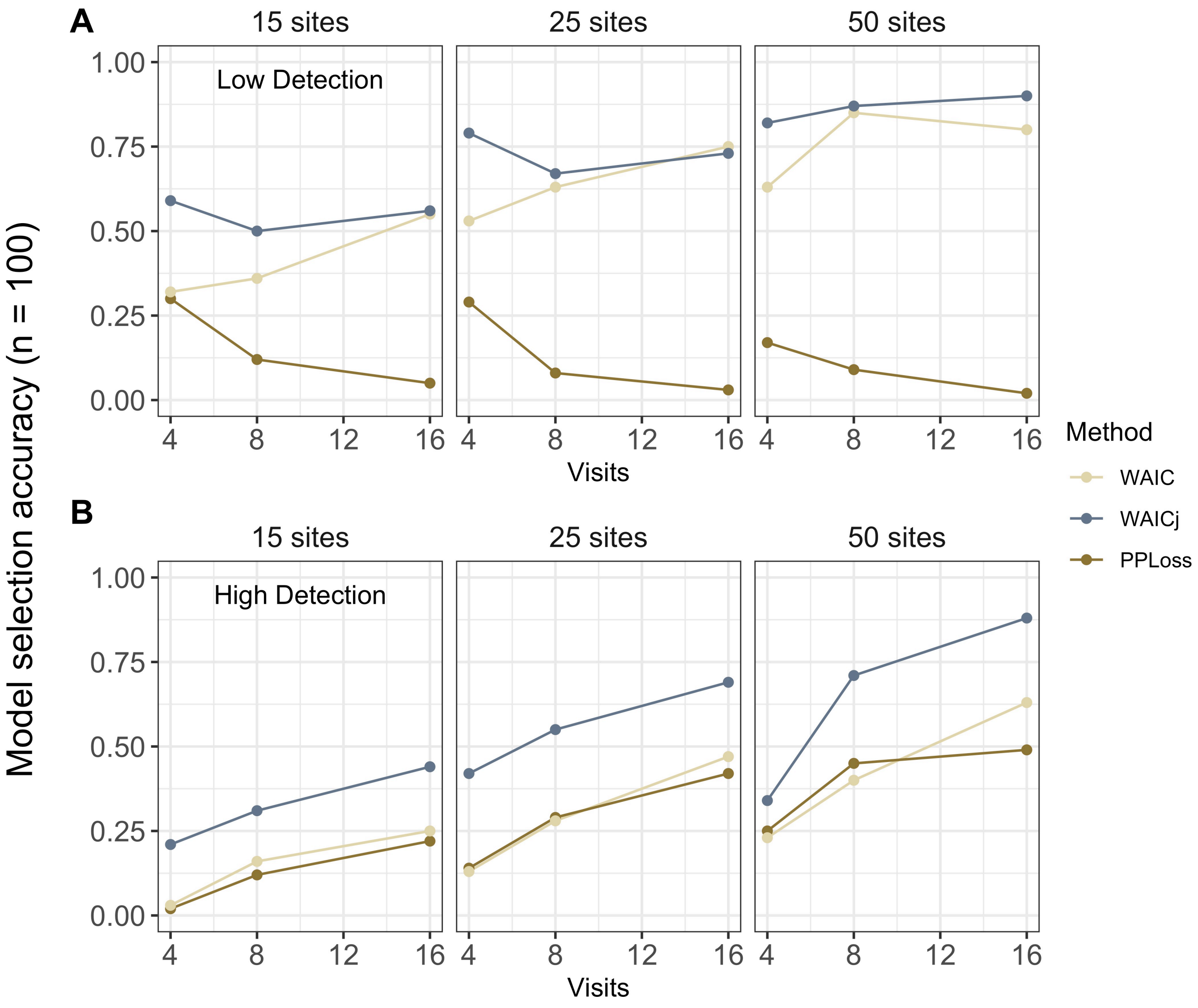

Figure S1: Proportion of simulations (n = 100) where the generating model was chosen as the top ranked model when simulated detection probability was close to 0 (A) or approaching 1 (B). Each simulation scenario was analyzed 100 times with data from 15, 25 or 50 sites and 4, 8, or 16 site visits per site.
